# Incidence of Newly Diagnosed Cancer After Cerebral Venous Thrombosis

**DOI:** 10.1001/jamanetworkopen.2024.58801

**Published:** 2025-02-10

**Authors:** Anita van de Munckhof, Jamie I. Verhoeven, Ilonca C. H. Vaartjes, Nick van Es, Frank-Erik de Leeuw, Jonathan M. Coutinho

**Affiliations:** 1 Department of Neurology, Amsterdam UMC, University of Amsterdam, Amsterdam, the Netherlands; 2 Department of Neurology, Research Institute for Medical Innovation, Radboud University Medical Center, Donders Institute for Brain, Cognition and Behavior, Nijmegen, the Netherlands; 3 Department of Epidemiology, Julius Center for Health Sciences and Primary Care, University Medical Center Utrecht, Utrecht, the Netherlands; 4 Department of Vascular Medicine, Amsterdam UMC, University of Amsterdam, Amsterdam, the Netherlands; 5 Amsterdam Cardiovascular Sciences, Pulmonary Hypertension, and Thrombosis, Amsterdam, the Netherlands

## Abstract

**Importance:**

Active cancer is a risk factor for cerebral venous thrombosis (CVT), but whether CVT is associated with occult cancer is unknown.

**Objective:**

To evaluate the incidence of newly diagnosed cancer after CVT.

**Design, Setting, and Participants:**

This population-based cohort study used data from the Dutch Hospital Discharge Registry from January 1, 1997, to July 1, 2020. Analyses were conducted between June 2023 and April 2024. Patients admitted with a first-ever CVT were included. Patients with a history of cancer or diagnosed with cancer during hospitalization for CVT were excluded.

**Exposure:**

CVT was identified using *International Classification of Diseases, Ninth Revision* (*ICD-9*) and *International Statistical Classification of Diseases and Related Health Problems, Tenth Revision* (*ICD-10*) codes.

**Main Outcomes and Measures:**

The main outcome was cumulative incidence of cancer after CVT. Standardized incidence ratios (SIRs) were calculated relative to a matched cohort from the general population using the Netherlands Cancer Registry.

**Results:**

A total of 2649 patients with CVT (median [IQR] age, 44.5 [30.7-56.4] years; 1856 [70.1%] female) were included. Cancer was diagnosed in 119 patients during a median (IQR) follow-up of 4.7 (1.9-8.9) years, of whom 29 (24.4%) had hematologic cancer. The cumulative incidence of cancer was 5.9% (95% CI, 4.8%-7.2%) after 10 years and was highest in men aged 50 years or older (13.5%; 95% CI, 9.1%-18.7%). Patients with CVT had an increased rate of cancer compared with the reference cohort during the entire follow-up, but the difference narrowed over time (SIRs of 3.35 [95% CI, 2.41-4.55] and 1.40 [95% CI, 1.14-1.69] at 1 and 10 years, respectively). The rate was increased both in patients younger than 50 years (SIRs of 6.70 [95% CI, 3.97-10.59] and 1.72 [95% CI, 1.24-2.34] at 1 and 10 years, respectively) and those 50 years or older (SIRs of 2.41 [95% CI, 1.53-3.62] and 1.25 [95% CI, 0.96-1.60] at 1 and 10 years, respectively), as well as in male patients (SIRs of 3.59 [95% CI, 2.16-5.61] and 1.69 [95% CI, 1.25-2.23] at 1 and 10 years, respectively) and female patients (SIRs, 3.17 [95% CI, 1.99-4.80] and 1.22 [95% CI, 0.92-1.58] at 1 and 10 years, respectively).

**Conclusions and Relevance:**

This cohort study of patients with CVT found an increased risk of cancer during follow-up regardless of age or sex. Men 50 years or older had the highest absolute risk, whereas younger patients had the highest relative risk. Physicians should be vigilant for signs of cancer after CVT, and further research on screening for cancer after CVT is warranted.

## Key Points

**Question:**

What is the incidence of newly diagnosed cancer after a first episode of cerebral venous thrombosis (CVT)?

**Findings:**

In this population-based cohort study of 2649 patients with CVT, the risk of new cancer was increased 3-fold compared with the general population in the first year after CVT and remained increased up to 10 years after CVT. The absolute risk of cancer was highest in men 50 years or older (13.5% after 10 years) but was relatively highest among patients younger than 50 years.

**Meaning:**

Patients with CVT have an increased risk of cancer; therefore, physicians should be vigilant for signs of cancer during follow-up.

## Teaser

This cohort study evaluates the incidence of newly diagnosed cancer in patients with cerebral venous thrombosis relative to the general population.

## Introduction

Cerebral venous thrombosis (CVT) is an uncommon cause of stroke, with an incidence of 1 to 2 per 100 000 person-years.^1,2^ As with other locations of thrombosis, active cancer is a well-known risk factor for CVT.^3,4,5,6^ Patients with cancer have an approximately 5-fold increased risk of CVT compared with patients without cancer, and this risk is highest within the first year after cancer diagnosis.^5^ Approximately 5% to 10% of patients with CVT have a history of cancer at the time of CVT diagnosis.^5,7,8^

It is unknown whether CVT is also associated with occult cancer (ie, cancer that is already present but has not yet been diagnosed at time of the CVT) or with an increased risk of developing cancer during follow-up. In the absence of data on this topic, current CVT guidelines do not recommend screening for cancer after CVT.^8,9^ Two population-based studies on the risk of new cancer after CVT diagnosis have recently been published.^10,11^ In a Finnish study,^10^ 13 of 589 patients with CVT (2.2%) had a new cancer diagnosis during 2-year follow-up, with the highest incidence in patients older than 50 years. In a Danish study,^11^ 43 of 811 patients with CVT (5.3%) had a new cancer diagnosis during a median follow-up of 6.2 years. Patients with CVT had an increased risk of cancer in the first months after CVT diagnosis, but overall, the incidence rate did not differ from the general population. In both studies, however, the sample size and the number of events during follow-up were small, resulting in uncertainty in the outcome estimates.

More data are necessary to better estimate how frequently patients are diagnosed with new cancer in the years following a first episode of CVT and to identify which groups of patients are at highest risk. We assessed the incidence of newly diagnosed cancer in the 10 years following a first CVT diagnosis and compared this with the incidence in a reference cohort from the general population.

## Methods

### Study Design

We performed a nationwide registry- and population-based cohort study of patients admitted to a hospital in the Netherlands with a first episode of CVT. Patients were identified using the Dutch Hospital Discharge Registry (HDR) linked to the Dutch Personal Records Database and the Dutch National Cause of Death Registry, as previously described.^12,13,14^ Data in the Dutch HDR and National Cause of Death Registry are collected by Statistics Netherlands and can be made available on request.^15^ Data were available from January 1, 1995, to December 31, 2020. This study was performed according to the guidelines of the medical ethical review board Oost-Nederland. No patients were directly recruited or actively involved, and ethical approval or individual patient consent was therefore not required according to Dutch law. This study followed the Strengthening the Reporting of Observational Studies in Epidemiology (STROBE) and Reporting of Studies Conducted Using Observational Routinely Collected Data (RECORD) reporting guidelines.

### Patient Selection

Patients 15 years or older with a first-registered episode of CVT between January 1, 1997, and July 1, 2020, were identified using *International Classification of Diseases, Ninth Revision* (*ICD-9*) and *International Statistical Classification of Diseases and Related Health Problems, Tenth Revision* (*ICD-10*) codes (eTable 1 in Supplement 1). We selected patients from 1997 to have at least 2 years of medical history available to have more certainty that the patient did not have a history of CVT or cancer. We included patients up to July 2020 to allow for at least 6 months of follow-up for all patients. Both primary (main) and secondary discharge diagnoses were used. Patients with a history of cancer and patients diagnosed with cancer during the same hospitalization as for the index CVT were excluded. The Charlson Comorbidity Index (CCI) was calculated at baseline. The CCI score is based on 19 medical conditions, such as diabetes and congestive heart failure, to classify burden of disease. A score of zero indicates that none of these comorbidities are present.^16^

### Definitions and Outcomes

The primary outcome was the cumulative incidence of cancer of any type within 10 years after the index CVT. We identified new cancer diagnoses during follow-up through corresponding *ICD-9* and *ICD-10* codes in the HDR (eTable 2 in Supplement 1).^14^ Nonmelanoma skin cancers were excluded.^17^ Patients were followed up from the index CVT until they reached 10 years of follow-up without an event, were diagnosed with cancer, reached December 31, 2020, died, or were lost to follow-up, whichever came first. If a patient had multiple cancers during follow-up, only the first diagnosed cancer was included in the analyses. Patients were considered lost to follow-up after emigration or loss of a unique linkage key. Until 2013, the personal linkage key was a combination of date of birth, sex, and postal code. After 2013, the linkage key was the Dutch Personal Identification Number. We stratified the cumulative incidence of new cancer by age at CVT diagnosis (<50 years and ≥50 years) and sex.

We compared the incidence rate of newly diagnosed cancer in patients with CVT with the incidence rate in a reference cohort from the general population by calculating standardized incidence ratios (SIRs). The reference cohort was selected from the general population by matching 5-year age groups, sex, and calendar year to the CVT cohort. The incidence rate of new cancer in the reference cohort was calculated using data from the Netherlands Cancer Registry. This registry is owned by Netherlands Comprehensive Cancer Organisation and contains reliable data of all patients with newly diagnosed cancer in the Netherlands.^18^ Cancer diagnoses in this registry are identified through the national pathology database, hospital data, and hematology laboratories. Data were available until 2021. We calculated SIRs for new cancer in the overall cohort and in subgroups based on age and sex. SIRs were also calculated for different types of cancer (eTable 2 in Supplement 1). In a post hoc analysis, we calculated the cumulative incidence and SIR for new hematologic and nonhematologic cancers separately.

Two additional subgroup analyses were conducted to investigate which specific group of patients was at highest risk of new cancer during follow-up. First, outcomes were calculated for male patients 50 years or older because we expected the incidence of cancer to be highest in this subgroup of patients with CVT. Second, we shifted the cut-off for the age-stratified analysis to 60 years instead of 50 years.

### Statistical Analysis

Data analyses were conducted between June 2023 and April 2024. We calculated the cumulative incidence of new cancer after CVT using the cumulative incidence function.^19^ Death from any cause was considered a competing risk.^20,21^ The Gray test was used to compare the subgroups.^22^ A 2-sided *P* < .05 was considered statistically significant. The follow-up duration in person-years was determined to calculate the SIRs. We assumed a Poisson distribution to calculate 95% CIs. Data analysis was performed using R, version 3.6.2 (R Foundation), with the packages rateratio.test, survival, survminer, and cmprsk; and Prism, version 5.03 (GraphPad Software). Incomplete or missing hospital admissions (ranging from 0%-18%) were not imputed. In a sensitivity analysis, we excluded patients diagnosed with cancer within 4 weeks after CVT diagnosis to correct for potential temporal coding errors or highly suspected cancers confirmed shortly after CVT diagnosis. If the number of events in any of the (sub)groups was less than 10, we were not allowed to disclose exact values to protect the privacy of individual patients.

## Results

**Figure 1.**
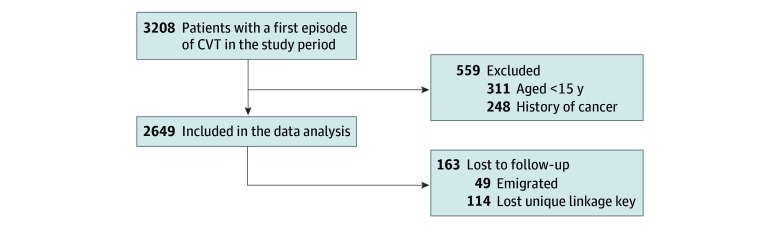
Flowchart of Patient Selection and Follow-Up CVT indicates cerebral venous thrombosis.

We identified 3208 patients admitted with CVT (Figure 1), of whom 248 (7.7%) were excluded because of a history of cancer and 311 (9.7%) because they were younger than 15 years. Of the 2649 included patients, 1856 (70.1%) were female and 793 (29.9%) were male (Table 1). The median (IQR) age at CVT diagnosis was 44.5 (30.7-56.4) years. The median (IQR) follow-up duration was 4.7 (1.9-8.9) years. During follow-up, 402 patients (15.2%) died, 114 (4.3%) lost their unique linkage key, and 49 (1.8%) emigrated.

**Table 1.** Baseline Characteristics of Patients With CVT

Characteristic	No. (%)^a^			*P* value
	Overall cohort (N = 2649)	Patients with cancer during follow-up (n = 119)	Patients without cancer during follow-up (n = 2530)	
Sex				
Female	1856 (70.1)	68 (57.1)	1788 (70.7)	.002
Male	793 (29.9)	51 (42.9)	742 (29.3)	
Age at time of CVT diagnosis, median (IQR), y	44.5 (30.7-56.4)	56.4 (45.6-63.9)	43.9 (30.3-55.5)	<.001
Age group, y				
≥50	961 (36.3)	70 (58.8)	891 (35.2)	<.001
≥60	554 (20.9)	48 (40.3)	506 (20.0)	<.001
Charlson Comorbidity Index score				
0	2236 (84.4)	96 (80.7)	2140 (84.6)	<.001
≥1	413 (15.6)	23 (19.3)	390 (15.4)	
Mortality during follow-up	402 (15.2)	66 (55.5)	336 (13.3)	<.001
Follow-up duration, median (IQR), y	4.7 (1.9-8.9)	2.2 (0.4-6.0)	4.8 (2.0-9.1)	<.001

Abbreviation: CVT, cerebral venous thrombosis.

^a^ Unless otherwise indicated.

Cancer was diagnosed in 119 patients (4.5%) during follow-up. The patients’ characteristics are provided in Table 1. Hematologic cancers (29 of 119 [24.4%]), digestive tract cancers (24 of 119 [20.2%]), and breast cancer (16 of 119 [13.4%]) were most commonly diagnosed during follow-up. The median (IQR) time from CVT to cancer diagnosis was 0.1 (0.0-3.0) years for hematologic cancers, 4.2 (1.6-6.0) years for digestive tract cancers, and 5.3 (4.1-8.3) years for breast cancer (Table 2).

**Table 2.** Types of Malignant Cancers Newly Diagnosed During Follow-Up After a First-Ever Episode of CVT

Cancer type	No. (%) of cancers	Time from CVT diagnosis to cancer diagnosis, median (IQR), y
All types	119 (100)	2.5 (0.6-7.2)
Hematologic	29 (24.4)	0.1 (0.0-3.0)
Other	25 (21.0)	3.1 (1.1-7.8)
Digestive tract	24 (20.2)	4.2 (1.6-6.0)
Breast	16 (13.4)	5.3 (4.1-8.3)
Lung	13 (10.9)	2.3 (0.0-7.6)
Central nervous system	12 (10.1)	0.5 (0.2-1.8)

Abbreviation: CVT, cerebral venous thrombosis.

### Cumulative Incidence

**Figure 2.**
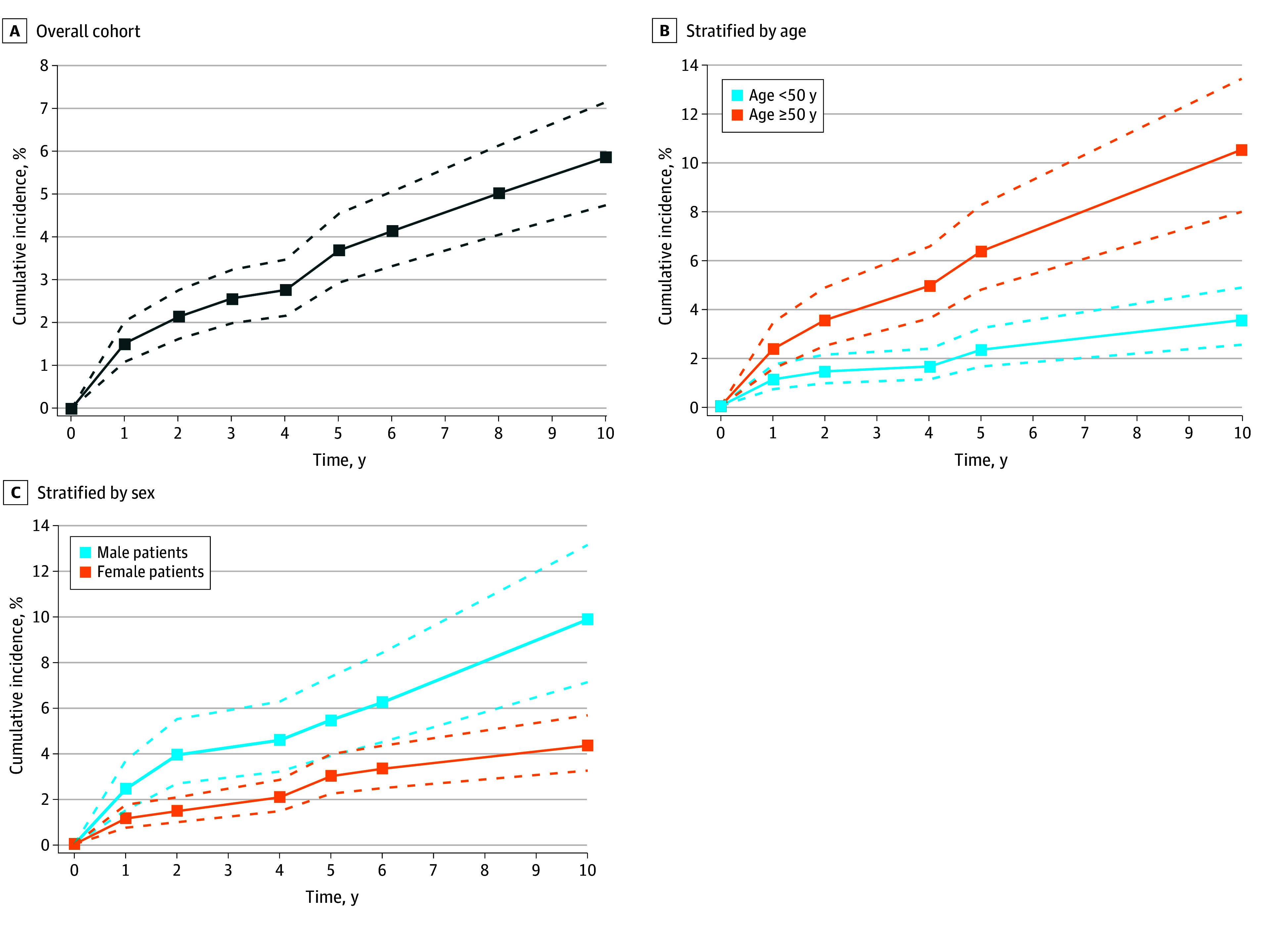
Cumulative Incidence of Newly Diagnosed Cancer in Patients With Cerebral Venous Thrombosis The dashed lines represent the 95% CIs.

In the overall cohort, the cumulative incidence of new cancer was 1.5% (95% CI, 1.1%-2.1%) after 1 year, 2.2% (95% CI 1.6%-2.8%) after 2 years, and 5.9% (95% CI, 4.8%-7.2%) after 10 years (Figure 2A). In patients younger than 50 years, the cumulative incidence of new cancer (1.1% [95% CI, 0.7%-1.7%] after 1 year and 3.5% [95% CI, 2.5%-4.8%] after 10 years) was lower compared with patients 50 years or older (2.3% [95% CI, 1.5%-3.4%] after 1 year and 10.5% [95% CI, 7.9%-13.4%] after 10 years) (*P* < .001) (Figure 2B). The cumulative incidence of new cancer was higher in male patients (2.4% [95% CI, 1.5%-3.7%] after 1 year and 9.9% [95% CI, 7.1%-13.1%] after 10 years) than in female patients (1.1% [95% CI, 0.7%-1.7%] after 1 year and 4.3% [95% CI, 3.2%-5.6%] after 10 years) (*P* < .001) (Figure 2C).

### Standardized Incidence Ratios

**Figure 3.**
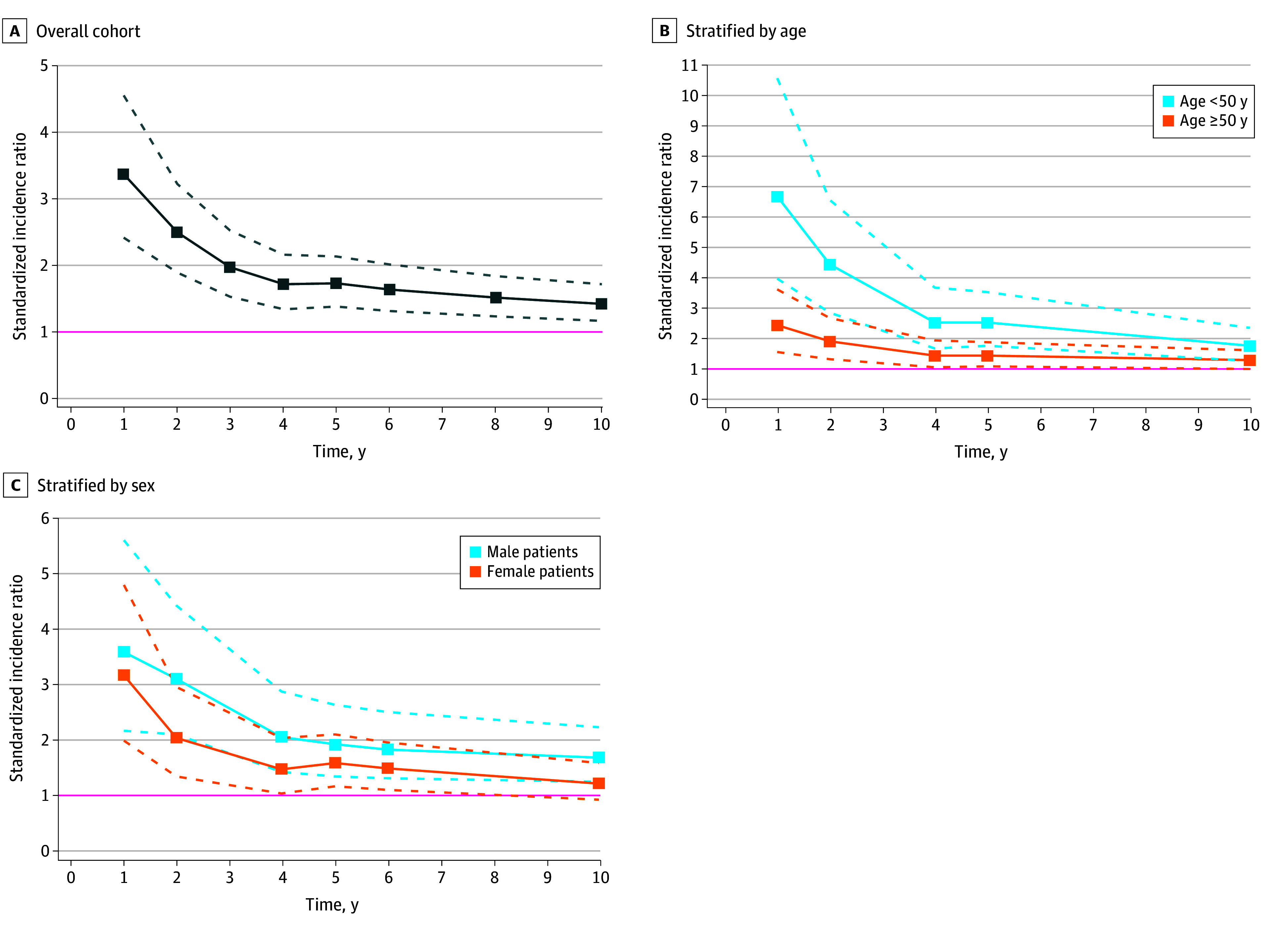
Standardized Incidence Ratios for Newly Diagnosed Cancer in Patients With Cerebral Venous Thrombosis Compared With a Reference Cohort The dashed lines represent the 95% CIs. The pink lines are reference lines for which the incidence rate in the cerebral venous thrombosis cohort equals the incidence rate in the reference cohort. Standardized incidence ratios above 1.0 indicate that the incidence rate of new cancer is higher in patients with cerebral venous thrombosis compared with the matched reference cohort.

Patients with CVT had an increased rate of cancer compared with the reference cohort for the entire duration of follow-up (SIRs of 3.35 [95% CI, 2.41-4.55] at 1 year and 1.40 [95% CI, 1.14-1.69] at 10 years; *P* < .001 for both) (Figure 3A). The incidence rate was increased for patients younger than 50 years (SIRs of 6.70 [95% CI, 3.97-10.59] and 1.72 [95% CI, 1.24-2.34] at 1 and 10 years, respectively) and in patients 50 years or older (SIRs of 2.41 [95% CI, 1.53-3.62] and 1.25 [95% CI, 0.96-1.60] at 1 and 10 years, respectively) (Figure 3B). In addition, the incidence rate of cancer was increased for both male and female patients with CVT (SIRs of 3.59 [95% CI, 2.16-5.61] and 1.69 [95% CI, 1.25-2.23] at 1 and 10 years for male patients and 3.17 [95% CI, 1.99-4.80] and 1.22 [95% CI, 0.92-1.58] at 1 and 10 years for female patients, respectively) (Figure 3C).

### Subgroup Analyses

The characteristics of patients with CVT with new hematologic vs nonhematologic cancer during follow-up are provided in eTable 3 in Supplement 1. The cumulative incidence of hematologic cancer was lower than for nonhematologic cancer (1.4% [95% CI, 0.9%-2.1%] vs 4.5% [95% CI, 3.5%-5.7%] at 10 years). The SIR was higher for hematologic cancer (17.76 [95% CI, 10.53-28.07] at 1 year and 4.61 [95% CI, 3.07-6.67] at 10 years) than for nonhematologic cancer (2.05 [95% CI, 1.30-3.08] at 1 year and 1.12 [0.88-1.40] at 10 years) (eTable 4 in Supplement 1). For the other prespecified subtypes of cancer, the number of events was too small to be disclosed.

In the subgroup analysis of male patients 50 years or older, 444 of 2649 patients (16.8%) were analyzed. The cumulative risk of new cancer in this group was 13.5% (95% CI, 9.1%-18.7%) at 10 years (eFigure 1A in Supplement 1). The SIRs for new cancer were 2.61 (95% CI, 1.35-4.57) and 1.19 (95% CI, 0.80-1.70) at 1 and 10 years, respectively (eFigure 1B in Supplement 1).

When comparing patients younger than 60 years with patients 60 years or older, we found a cumulative incidence of 1.3% (95% CI, 0.8%-1.8%) vs 4.2% (95% CI, 3.4%-5.1%) at 1 year and 4.0% (95% CI, 3.0%-5.3%) vs 6.1% (95% CI, 5.0%-7.3%) at 10 years, respectively. The SIRs for new cancer in patients younger than 60 years and 60 years or older were 5.29 (95% CI, 3.45-7.75) and 2.05 (95% CI, 1.15-3.38) at 1 year and 1.43 (95% CI, 1.09-1.86) and 1.36 (95% CI, 1.00-1.81) at 10 years, respectively.

### Sensitivity Analysis

After exclusion of 20 patients diagnosed with cancer within 4 weeks of their CVT diagnosis, 2629 patients were analyzed. Baseline sex and age of this cohort were comparable to the overall cohort (eTable 5 in Supplement 1). The cumulative risk of new cancer was 0.8% (95% CI, 0.5%-1.2%) at 1 year and 5.2% (95% CI, 4.1%-6.4%) at 10 years (eFigure 2A in Supplement 1). The SIRs were 1.72 (95% CI, 1.06-2.63) at 1 year and 1.13 (95% CI, 0.91-1.40) at 10 years (eFigure 2B in Supplement 1).

## Discussion

The results of this large population-based cohort study suggest that patients with a first episode of CVT have an increased risk of a cancer diagnosis, in particular in the first year after CVT. The cumulative incidence of cancer was 5.9% after 10 years and was highest in men 50 years or older. The incidence rate of cancer was increased in patients with CVT compared with a matched reference cohort for the entire duration of follow-up, regardless of age and sex. The SIR was highest among patients younger than 50 years. Hematologic cancers, digestive tract cancers, and breast cancers were the most commonly diagnosed cancers.

In a study from Finland, 2.2% patients with CVT without a history of cancer were diagnosed with cancer during a 2-year follow-up period,^10^ which is similar to the cumulative incidence after 2 years in our study. A study from Denmark reported new cancer in 5.3% patients with CVT after a median follow-up of 6.2 years.^11^ In that study, the overall incidence rate of new cancer was not increased in patients with CVT compared with the general population (SIR, 1.04; 95% CI, 0.75-1.40). The authors did find a 7-fold increased risk of cancer in the first 3 months after CVT diagnosis, but the incidence ratio reversed after 1 year of follow-up (SIR, 0.76; 95% CI, 0.50-1.09). The risk of new cancer in our cohort, however, remained significantly elevated up to 10 years after the index CVT. Possible explanations for this difference could be the small number of events in the Danish cohort hampering detection of significant differences or the follow-up period of up to 20 years, which could neutralize the results. The Finnish study^10^ did not include a reference population, precluding direct comparisons of relative incidence rates.

We used *ICD-9* and *ICD-10* codes to identify patients with CVT. These codes have been shown to be reliable for identification of CVT from administrative data.^23,24,25^ In a study from the US, discharge *ICD-9* codes for CVT had a sensitivity of 77.8% and specificity of 92.7%.^24^ For *ICD-10* codes, the positive predictive value was 92% to 95% in studies from the United Kingdom and Canada but with a sensitivity of 42% for new CVT in the Canadian study.^23,25^ The Netherlands had a population between 15.6 million and 17.4 million people during the study period.^26^ With an estimated incidence of CVT of 1 to 2 per 100 000 persons per year, approximately 3870 to 7740 patients with CVT would be expected in the studied period, which is slightly higher than the number of patients in our study. Because hospital discharge data were used to identify patients, we did not capture patients who were never admitted to a hospital, although we expect these cases to be very rare. Another reason for the smaller number of patients in our cohort could be the moderate to low sensitivity of *ICD* codes for identifying CVT, missing or incomplete hospital admission records, or the fact that not all hospitals in the Netherlands contributed equally to the HDR during the study period.^27^ Although we may have missed some patients, baseline characteristics, including age, sex, and the proportion of patients with known cancer at the time of CVT diagnosis, are similar to previously published CVT cohorts,^2,7^ which suggests that our cohort is representative for the general CVT population.

New cancer diagnoses were also identified based on HDR data. We expect that most patients with cancer were admitted for treatment at some point during the disease, but the number of new cancer diagnoses in our cohort could be underestimated. In the reference cohort, however, cancers were identified using both hospital data and pathological data, which may have resulted in a higher, more accurate incidence rate of new cancer in the reference cohort compared with the CVT cohort. The temporal relationship between CVT and cancer cannot be determined with certainty based on our data. It is possible that in some cases, concomitant cancer diagnoses were not registered before or during hospitalization for the index CVT and are therefore wrongly considered as newly diagnosed cancers during follow-up. This issue could, for example, be reflected by the short interval from CVT to diagnosis of hematologic cancer. In the sensitivity analysis correcting for possible temporal misclassifications, a significantly increased rate of new cancer was still observed in the first 2 years after CVT diagnosis.

For cancers diagnosed relatively soon after CVT (eg, within 1 year), it is likely that the index CVT was a manifestation of occult cancer, considering the well-established pathophysiologic mechanisms underlying cancer-related thrombosis.^3,4,5,6^ However, for cancers diagnosed at a later stage, it is more uncertain whether these were occult cancers or whether CVT and cancer were otherwise associated. The interval from CVT to cancer diagnosis was longest for breast cancers and digestive tract cancers. CVT could be associated with these cancers through common risk factors, such as oral contraceptive use and inflammatory bowel disease.^28,29^ Nevertheless, the persistence of an increased risk of cancer for several years after a first episode of thrombosis has also been described for unprovoked venous thromboembolism.^30^ The high mortality among patients with newly diagnosed cancer in our study indicates that these cancers were clinically relevant. It is not known, however, whether earlier detection would improve outcomes of patients with CVT.

On the basis of our data, physicians should be alert to signs of cancer during follow-up of patients with CVT. However, this does not imply that patients with CVT should be actively screened for occult cancer, as this was not specifically examined in our study. Instead, the results of our study call for additional studies on this topic.^31^ Cost-effectiveness and burden on patients, for example, through an increased radiation load or increased number of investigations, should be taken into account when evaluating screening for occult cancer. In addition, different screening strategies should be considered. In unprovoked venous thromboembolism, for example, extensive screening for occult cancer with comprehensive computed tomography or positron emission tomography–computed tomography did not result in a higher yield of cancer or show a clinical benefit^32,33,34^; however, limited screening with a thorough medical history and physical examination, laboratory investigation, and chest radiography is recommended.^35^ Given the higher absolute risk, the chance of detecting a new cancer would be highest in male patients with CVT 50 years or older.

### Limitations

Our study has several limitations. First, details on risk factors for CVT or cancer, clinical symptoms at presentation, and anticoagulation status during follow-up were not available. These data could be valuable to better identify patients at highest risk of new cancer during follow-up. Second, the characteristics and staging of the newly diagnosed cancers were not available, which would be valuable to assess the treatability and clinical relevance of these cancers. Third, until 2013, linkage between the different registries was based on a linkage key, which potentially could have resulted in incorrect linkage between events and persons. Fourth, data were retrospectively collected from population-based registries that were not specifically designed to answer our research question. The accuracy of *ICD-9* and *ICD-10* codes for CVT and cancer was not again validated for use in the HDR, potentially resulting in a misclassification bias. The CCI scores derived from these administrative data could have underestimated the presence of comorbidities in the CVT cohort. In addition, the HDR does not contain detailed patient characteristics, resulting in unmeasured confounding. Fifth, despite the nationwide coverage and long study period, the number of patients with a new cancer diagnosis after CVT was still limited, which restricted the stratification of the incidence per cancer subtype.

## Conclusions

In this cohort study of patients with CVT, we found an increased risk of a new cancer diagnosis compared with a reference cohort from the general population, regardless of age or sex. The absolute risk of cancer was highest in men 50 years or older, whereas the risk was relatively highest in younger patients. Physicians should be vigilant for signs of cancer during follow-up of patients with CVT. These results support the need for further research on screening for occult cancer after CVT.

## Notes

Supplement 1. **eTable 1.** ICD-9 and ICD-10 codes used to identify cerebral venous thrombosis **eTable 2.** ICD codes used to identify new invasive, malignant cancer during follow-up **eTable 3.** Characteristics of patients with CVT who were newly diagnosed with hematologic versus nonhematologic cancer during follow-up **eTable 4.** Cumulative incidence and SIR stratified for new hematologic and nonhematologic cancer diagnoses after CVT **eTable 5.** Baseline characteristics after exclusion of patients who were diagnosed with cancer within four weeks of CVT diagnosis **eFigure 1.** Cumulative incidence and SIR for any first-ever cancer diagnosis in male patients with CVT who are 50 years or older at the time of CVT diagnosis **eFigure 2.** Cumulative incidence and SIR for any first-ever cancer diagnosis in patients with CVT after exclusion of patients who were diagnosed with cancer within four weeks of CVT diagnosis

Supplement 2.
Data Sharing Statement
