# Supplementary material for: Incidence of Newly Diagnosed Cancer After Cerebral Venous Thrombosis
Source: JAMA Netw Open. 2025 Feb 10;8(2):e2458801. doi: 10.1001/jamanetworkopen.2024.58801 (PMC11811797; doi:10.1001/jamanetworkopen.2024.58801)
Supplement: Supplement 1. — eTable 1. ICD-9 and ICD-10 codes used to identify cerebral venous thrombosis eTable 2. ICD codes used to identify new invasive, malignant cancer during follow-up eTable 3. Characteristics of patients with CVT who were newly diagnosed with hematologic versus nonhematologic cancer during follow-up eTable 4. Cumulative incidence and SIR stratified for new hematologic and nonhematologic cancer diagnoses after CVT eTable 5. Baseline characteristics after exclusion of patients who were diagnosed with cancer within four weeks of CVT diagnosis eFigure 1. Cumulative incidence and SIR for any first-ever cancer diagnosis in male patients with CVT who are 50 years or older at the time of CVT diagnosis eFigure 2. Cumulative incidence and SIR for any first-ever cancer diagnosis in patients with CVT after exclusion of patients who were diagnosed with cancer within four weeks of CVT diagnosis [file jamanetwopen-e2458801-s001.pdf]

Supplemental Online Content

van de Munckhof A, Verhoeven JI, Vaartjes ICH, van Es N, de Leeuw FE, Coutinho JM. Incidence of newly diagnosed cancer after cerebral venous thrombosis. *JAMA Netw Open*. Published online February 10, 2025. doi:10.1001/jamanetworkopen.2024.58801

eTable 1. ICD-9 and ICD-10 codes used to identify cerebral venous thrombosis

eTable 2. ICD codes used to identify new invasive, malignant cancer during follow-up

eTable 3. Characteristics of patients with CVT who were newly diagnosed with hematologic versus nonhematologic cancer during follow-up

eTable 4. Cumulative incidence and SIR stratified for new hematologic and nonhematologic cancer diagnoses after CVT

eTable 5. Baseline characteristics after exclusion of patients who were diagnosed with cancer within four weeks of CVT diagnosis

eFigure 1. Cumulative incidence and SIR for any first-ever cancer diagnosis in male patients with CVT who are 50 years or older at the time of CVT diagnosis

eFigure 2. Cumulative incidence and SIR for any first-ever cancer diagnosis in patients with CVT after exclusion of patients who were diagnosed with cancer within four weeks of CVT diagnosis

This supplemental material has been provided by the authors to give readers additional information about their work.

© 2025 van de Munckhof A et al. *JAMA Netw Open*.

eTable 1. ICD-9 and ICD-10 codes used to identify cerebral venous thrombosis.

Classification	Code	Description
ICD-9	325.0	Cerebral sinovenous thrombosis, excluding nonpyogenic cases and cases associated with pregnancy and the puerperium
	437.6	CVT of nonpyogenic origin
	671.5	CVT complicating pregnancy, childbirth, or the puerperium
ICD-10	G08.X	Intracranial and intraspinal phlebitis and thrombophlebitis
	I67.6	Non-pyogenic thrombosis of intracranial venous system
	I63.6	Cerebral infarction due to CVT, non-pyogenic
	O22.5	CVT in pregnancy
	O87.3	CVT in the puerperium

CVT = cerebral venous thrombosis; ICD-9 = International Statistical Classification of Diseases and Related Health Problems Ninth Revision; ICD-10 = International Statistical Classification of Diseases and Related Health Problems Tenth Revision.

© 2025 van de Munckhof A et al. *JAMA Netw Open*.

eTable 2. ICD codes used to identify new invasive, malignant cancer during follow-up.

Classification	Cancer group	Code	Description
ICD-9	Breast	174, 175, 198.81	Female and male breast
	Central nervous system	191, 192, 198.3, 198.4	Brain, other and unspecified parts of nervous system
	Digestive tract	150-157, 159, 197.4-197.8	Digestive organs
	Hematologic	200-209	Lymphatic and hematopoietic tissue
	Lung	162, 197.0-197.3	Trachea, bronchus, and lung
	Urogenital tract	179-189, 198.0, 198.1, 198.6, 198.82	Genitourinary organs
	Other	140-149	Lip, oral cavity, and pharynx
		158	Retroperitoneum and peritoneum
		160-165	Respiratory and intrathoracic organs (excl. trachea, bronchus, and lung)
		170-172, 176, 198.5	Bone and articular cartilage, connective and other soft tissue, malignant melanoma of skin, Kaposi's sarcoma
		190	Eye
		193, 194, 198.7	Thyroid gland, other endocrine glands and related structures
		195, 196, 198.89, 199	Other and ill-defined sites or without specification of site
ICD-10	Breast	C50	Breast
	Central nervous system	C70, C71, C72	Meninges, brain, spinal cord, cranial nerves and other parts of central nervous system
	Digestive tract	C15-C26	Digestive organs
	Hematologic	C81-C96	Malignant neoplasms, stated or presumed to be primary, of lymphoid, haematopoietic and related tissue
	Lung	C33, C34	Trachea, bronchus, and lung
	Urogenital tract	C51-C58	Female genital organs
		C60-C63	Male genital organs
		C64-C68	Urinary tract
	Other	C00-C14	Lip, oral cavity, and pharynx
		C30-C32, C35-C39	Respiratory and intrathoracic organs (excl. trachea, bronchus, and lung)
		C40-C41	Bone and articular cartilage
		C43	Malignant melanoma of skin
		C45-C49	Mesothelial and soft tissue
		C69	Eye and adnexa
		C73-C75	Thyroid and other endocrine glands
		C76, C80	Other and ill-defined sites or without specification of site

ICD-9 = International Statistical Classification of Diseases and Related Health Problems Ninth Revision; ICD-10 = International Statistical Classification of Diseases and Related Health Problems Tenth Revision. Please note that non-melanoma skin cancers (ICD-9 codes 173 [Other and unspecified malignant neoplasm of skin] and 198.2 [Secondary malignant neoplasm of skin] and ICD-10 code C44 [Other malignant neoplasms of skin]) are excluded from the analysis. The group of hematologic cancers does not include myeloproliferative neoplasms (ICD-9 codes 238.4 and 238.7; ICD-10 codes D45 and D46).

© 2025 van de Munckhof A et al. *JAMA Netw Open*.

eTable 3. Characteristics of patients with CVT who were newly diagnosed with hematologic versus nonhematologic cancer during follow-up.

Characteristic, No. (%)	Hematologic cancer during follow-up (N = 29)	Nonhematologic cancer during follow-up (N = 90)
Sex		
Female	12 (41.4)	56 (62.2)
Male	17 (58.6)	34 (37.8)
Age at time of CVT diagnosis, median (IQR), y	57.4 (32.0-65.2)	56.2 (46.2-63.7)
Age >= 50 years	17 (58.6)	53 (58.9)
Mortality during follow-up	14 (48.3)	52 (57.8)
Follow-up duration, median (IQR), y	0.2 (0.2-3.1)	3.3 (1.1-7.5)

CVT = cerebral venous thrombosis; IQR = interquartile range.

© 2025 van de Munckhof A et al. *JAMA Netw Open*.

eTable 4. Cumulative incidence and SIR stratified for new hematologic and nonhematologic cancer diagnoses after CVT.

	Cumulative number of observed cancer diagnoses	Cumulative incidence, % (95% Confidence Interval)	SIR (95% Confidence Interval)
**Hematologic cancer**			
Overall cohort			
At 1 y	18	0.7% (0.42-1.06)	17.8 (10.53-28.07)
At 5 y	23	0.9% (0.60-1.37)	5.8 (3.68-8.72)
At 10 y	28	1.4% (0.93-2.13)	4.6 (3.07-6.67)
Age < 50 years			
At 1 y	9	0.5% (0.27-0.99)	39.9 (18.26-75.77)
Age >= 50 years			
At 1 y	9	0.9% (4.47-1.74)	11.4 (5.22-21.69)
Female			
At 1 y	8	0.4% (0.21-0.83)	15.6 (6.75-30.70)
Male			
At 1 y	10	1.3% (0.66-2.26)	20.0 (9.59-36.79)
**Nonhematologic cancer**			
Overall cohort			
At 1 y	22	0.8% (0.55-1.26)	2.1 (1.30-3.08)
At 5 y	60	2.8% (2.15-3.58)	1.3 (1.03-1.73)
At 10 y	77	4.5% (3.51-5.66)	1.1 (0.88-1.40)
Age < 50 years			
At 1 y	9	0.5% (0.27-1.00)	3.7 (1.67-6.94)
Age >= 50 years			
At 1 y	13	1.4% (0.77-2.28)	1.6 (0.87-2.68)
Female			
At 1 y	13	0.7% (0.41-1.19)	2.2 (1.19-3.66)
Male			
At 1 y	9	1.1% (0.57-2.10)	1.9 (0.86-3.57)

CVT = cerebral venous thrombosis; SIR = Standardized Incidence Ratio.

© 2025 van de Munckhof A et al. *JAMA Netw Open*.

eTable 5. Baseline characteristics after exclusion of patients who were diagnosed with cancer within four weeks of CVT diagnosis.

Characteristic, No. (%)	Total cohort (N = 2629)	CVT patients with cancer during follow-up (N = 99)	CVT patients without cancer during follow-up (N = 2530)
Female patients	1846 (70.2)	58 (58.6)	1788 (70.7)
Age at time of CVT diagnosis, median (IQR), y	44.5 (30.7-56.4)	57.0 (45.9-64.8)	43.9 (30.3-55.5)
Follow-up duration, median (IQR), y	4.8 (1.9-9.0)	4.0 (1.2-7.6)	4.8 (2.0-9.1)

CVT = cerebral venous thrombosis; IQR = interquartile range.

© 2025 van de Munckhof A et al. *JAMA Netw Open*.

eFigure 1. Cumulative incidence and SIR for any first-ever cancer diagnosis in male patients with CVT who are 50 years or older at the time of CVT diagnosis.

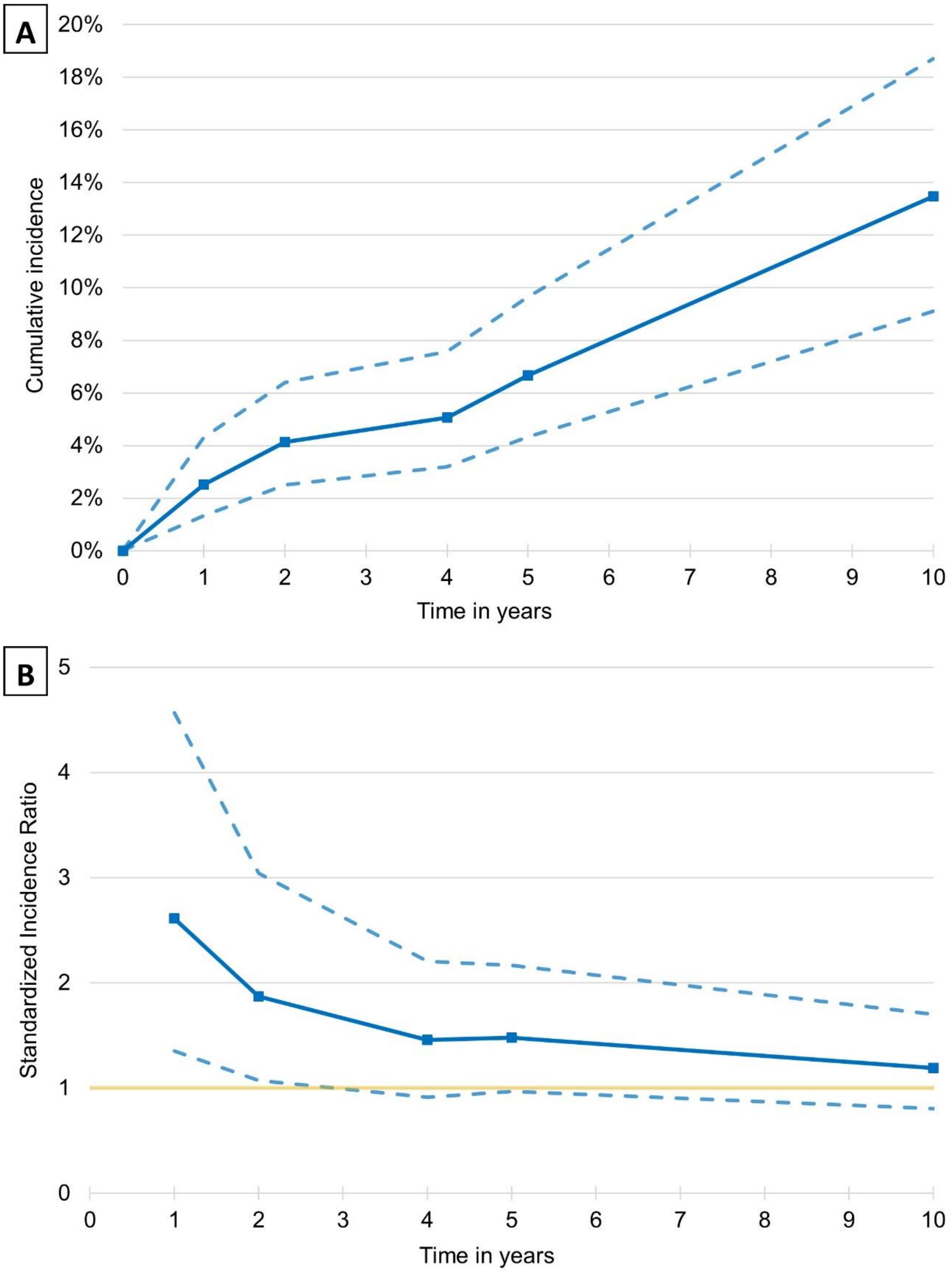

CVT = cerebral venous thrombosis; SIR = Standardized Incidence Ratio.

Panel A = Cumulative incidence of newly diagnosed cancer over time. Panel B = SIR for newly diagnosed cancer in male patients with CVT 50 years or older compared with a reference cohort from the general population.

The dashed blue lines represent the upper- and lower 95% Confidence Interval values. The yellow line is a reference line for which the incidence rate in the CVT cohort equals the incidence rate in the reference cohort.

© 2025 van de Munckhof A et al. *JAMA Netw Open*.

eFigure 2. Cumulative incidence and SIR for any first-ever cancer diagnosis in patients with CVT after exclusion of patients who were diagnosed with cancer within four weeks of CVT diagnosis.

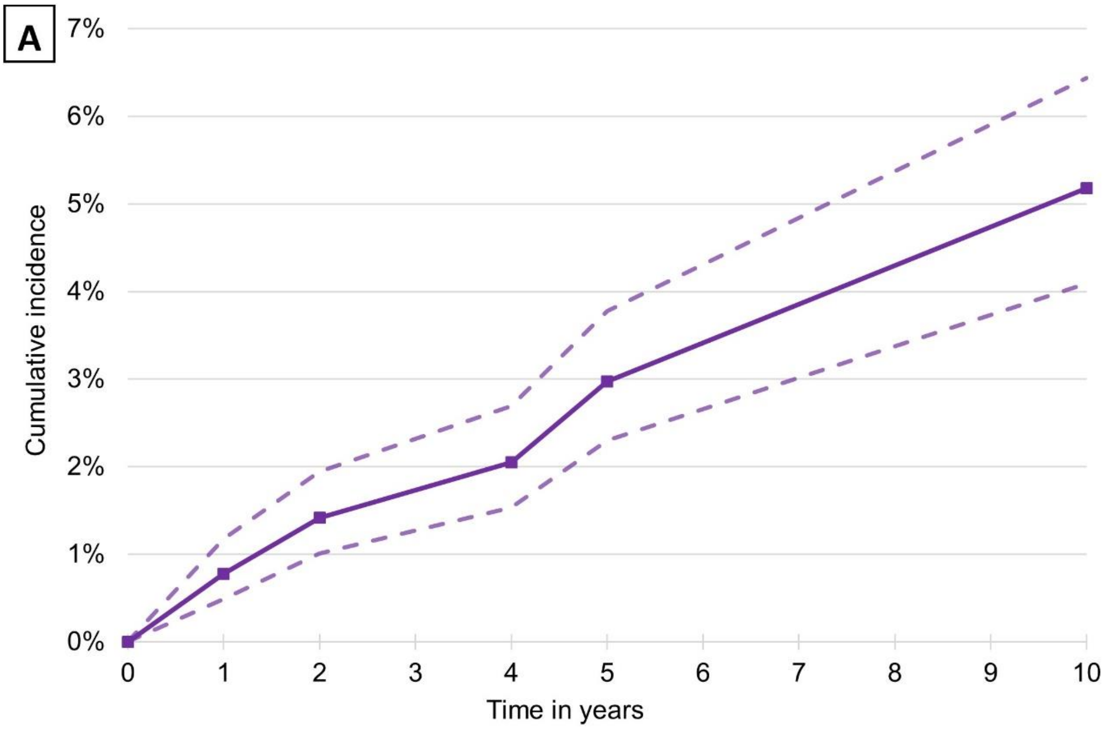

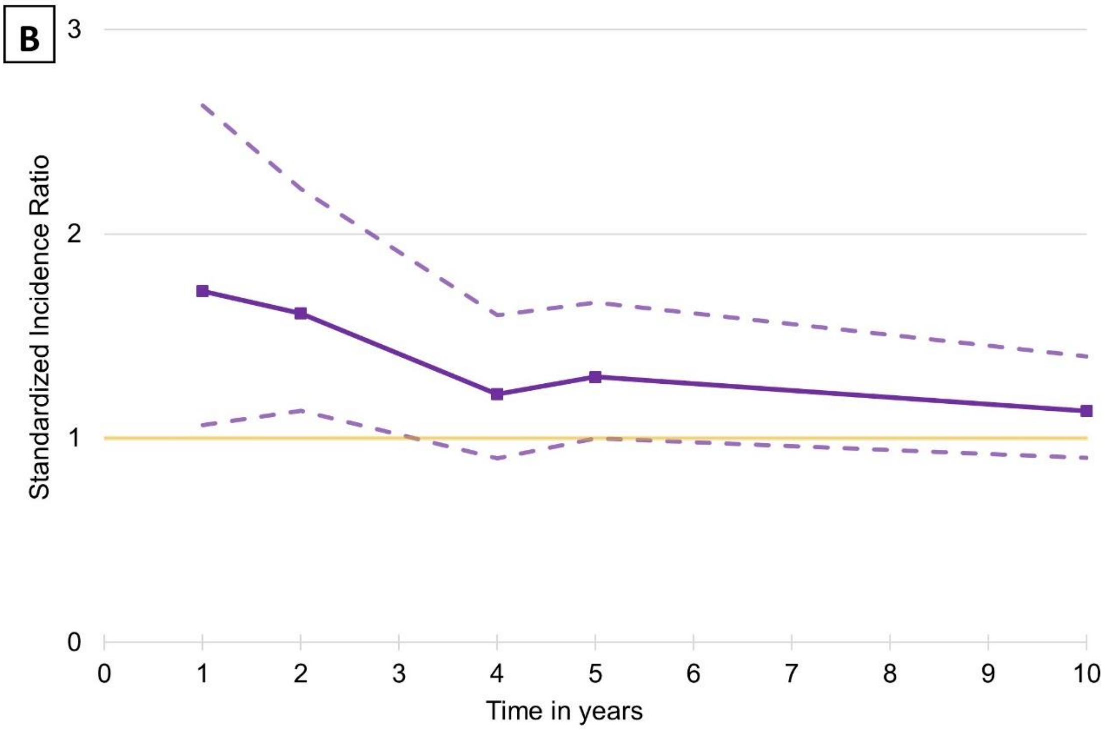

CVT = cerebral venous thrombosis; SIR = Standardized Incidence Ratio.

Panel A = Cumulative incidence of newly diagnosed cancer over time. Panel B = SIR for newly diagnosed cancer in patients with CVT compared with a reference cohort from the general population.

The dashed purple lines represent the upper- and lower 95% Confidence Interval values. The yellow line is a reference line for which the incidence rate in the CVT cohort equals the incidence rate in the reference cohort.

© 2025 van de Munckhof A et al. *JAMA Netw Open*.
