# Supplementary material for: Incidence of Newly Diagnosed Cancer After Cerebral Venous Thrombosis
Source: JAMA Netw Open. 2025 Feb 10;8(2):e2458801. doi: 10.1001/jamanetworkopen.2024.58801 (PMC11811797; doi:10.1001/jamanetworkopen.2024.58801)
Supplement: Supplement 2. — Data Sharing Statement [file jamanetwopen-e2458801-s002.pdf]

Data Sharing Statement

van de Munckhof. Incidence of Newly Diagnosed Cancer After Cerebral Venous Thrombosis. *JAMA Netw Open*. Published February 10, 2025. doi:10.1001/jamanetworkopen.2024.58801

Data
Data available: No

Additional Information
Explanation for why data not available: Data in the Dutch Hospital Discharge Registry and National Cause of Death Registry are collected by Statistics Netherlands and can be made available upon request.
